# Network discovery with DCM

**DOI:** 10.1016/j.neuroimage.2010.12.039

**Published:** 2011-06-01

**Authors:** Karl J. Friston, Baojuan Li, Jean Daunizeau, Klaas E. Stephan

**Affiliations:** a The Wellcome Trust Centre for Neuroimaging, University College London, Queen Square, London WC1N 3BG, UK; b Laboratory for Social and Neural Systems Research, Dept. of Economics, University of Zurich, Switzerland; c College of Mechatronic Engineering and Automation, National University of Defense Technology, Changsha, Hunan, 410073, PR China

**Keywords:** Bayesian, Neuronal, Generalised Filtering, Dynamic Causal Modelling, fMRI, Random differential equations, Stochastic, Resting-state, Connectivity

## Abstract

This paper is about inferring or discovering the functional architecture of distributed systems using Dynamic Causal Modelling (DCM). We describe a scheme that recovers the (dynamic) Bayesian dependency graph (connections in a network) using observed network activity. This network discovery uses Bayesian model selection to identify the sparsity structure (absence of edges or connections) in a graph that best explains observed time-series. The implicit adjacency matrix specifies the form of the network (e.g., cyclic or acyclic) and its graph-theoretical attributes (e.g., degree distribution). The scheme is illustrated using functional magnetic resonance imaging (fMRI) time series to discover functional brain networks. Crucially, it can be applied to experimentally evoked responses (activation studies) or endogenous activity in task-free (resting state) fMRI studies. Unlike conventional approaches to network discovery, DCM permits the analysis of directed and cyclic graphs. Furthermore, it eschews (implausible) Markovian assumptions about the serial independence of random fluctuations. The scheme furnishes a network description of distributed activity in the brain that is optimal in the sense of having the greatest conditional probability, relative to other networks. The networks are characterised in terms of their connectivity or adjacency matrices and conditional distributions over the directed (and reciprocal) effective connectivity between connected nodes or regions. We envisage that this approach will provide a useful complement to current analyses of functional connectivity for both activation and resting-state studies.

## Research Highlights

►A new way of analysing effective connectivity. ►A way of searching over large sets of models (networks) using DCM. ►Identifies the best network or graph using Bayesian model evidence. ►Can be applied to resting state and activation studies.

## Introduction

Historically, Dynamic Causal Modelling (DCM) has been portrayed as a hypothesis-led approach to understanding distributed neuronal architectures underlying observed brain responses (Friston et al., 2003). Generally, competing hypotheses are framed in terms of different networks or graphs, and Bayesian model selection is used to quantify the evidence for one network (hypothesis) over another (Penny et al., 2004). However, in recent years, the number of models over which people search (the model-space) has grown enormously; to the extent that DCM is now used to discover the best model over very large model-spaces (e.g., Stephan et al., 2010; Penny et al., 2010). Here, we take this discovery theme one step further and throw away prior knowledge about the experimental causes of observed responses to make DCM entirely data-led. This enables network discovery using observed responses during both activation studies and (task-free) studies of autonomous or endogenous activity during the “resting state”. In what follows, we describe this scheme in terms of the underlying generative model, the construction of model-spaces and how these spaces are searched for the optimum model. In addition to covering the theoretical background to DCM discovery, we illustrate its application to an fMRI (attention to motion) dataset used previously in technical papers. In subsequent work, we will illustrate its application to resting-state fMRI data (Li et al., in preparation).

This paper comprises four sections. In the first, we describe the form of the DCM used in subsequent sections. This is exactly the same as the conventional DCM for fMRI but includes endogenous fluctuations, which are represented by random differential equations (Li et al., 2010). These equations can be regarded as a [bi]linear approximation to any nonlinear model of neuronal dynamics. In this paper, we take a closer look at what this linear approximation means, when considering endogenous fluctuations that arise from self-organised dynamics (e.g., Suckling et al., 2008; Honey et al, 2009). Having established the basic form of our model, we then turn to model inversion and consider briefly the distinction between deterministic and stochastic schemes. This distinction is important because stochastic or random fluctuations are inevitable, when modelling self-organised dynamics at a macroscopic scale. The second section deals with the construction of model-spaces and how each model within these spaces is evaluated or ‘scored’. The main focus of this section will be on efficient scoring and plausible constraints or priors on models that restrict the search to sensible subspaces. We consider the difficult problem of scoring very large numbers of models. To finesse this problem we use a proxy for the model evidence based upon the conditional density over the parameters of a fully connected network (the Savage–Dickey density ratio; Dickey, 1971). This section borrows several ideas from graph theory and connects them with Bayesian constructs in DCM. The resulting search scheme is used in Monte Carlo simulations to evaluate and validate the accuracy of model selection; i.e., network discovery. The third section applies these procedures to an empirical fMRI time-series, acquired under an attention to motion paradigm. This section illustrates the sorts of results that can be obtained and revisits some key questions about the functional architecture of hierarchies in the brain and the relative expression of top-down and bottom-up influences. We conclude with a brief discussion of the relevance of this scheme for determining neuronal architectures from measured brain responses in general, and its implications for the characterisation of fMRI time-series in particular.

## The generative model and its inversion

This section describes the causal or generative model for the specific fMRI application considered in this paper. The model is basically the same as the conventional DCM for fMRI; however, we will motivate the assumptions implicit in the usual approximation. This more detailed examination of DCM for fMRI discloses the central importance (and nature) of fluctuations in neurophysiologic states, which have been ignored in classical (deterministic) variants of Dynamic Causal Modelling.

We have introduced several schemes recently that accommodate fluctuations on hidden neuronal and other physiological states (Penny et al., 2005; Daunizeau et al, 2009; Friston et al., 2010; Li et al., 2010). This means that one can estimate hidden states generating observed data, while properly accommodating endogenous or random fluctuations. These become particularly important when modelling endogenous dynamics, which are itinerant (wandering) and sometimes ergodic (e.g., resting-state fMRI time-series). We will exploit these schemes to search over models that couple fluctuating dynamics in different parts of the brain. We first consider the generative model *per se* and then turn to its inversion or optimisation. Here, we take the opportunity to consider two alternative approaches to dealing with fluctuations in neuronal activity; the first is based upon Generalised Filtering for stochastic DCM described in Friston et al. (2010) and applied to fMRI in Li et al. (2010). The nature of these generalised schemes speaks to the fact that there is no real difference between hidden states and parameters in DCM; therefore, it should be possible to cast unknown fluctuations in neuronal states as unknown parameters. In fact, this approach was used in the pioneering work of Riera et al. (2004). We will address the implicit exchangeability of states and parameters by comparing stochastic DCM (Daunizeau et al., 2009; Li et al., 2010) with deterministic DCMs that model unknown fluctuations in neuronal states with a mixture of temporal basis functions.

### The generative model

DCM for fMRI rests on a generative model that has two components. The first is a neuronal model describing interactions (dependencies) in a distributed network of neuronal populations. The second component maps neuronal activity to observed hemodynamic responses. This component has been described in detail many times previously and rests on a hemodynamic model (subsuming the Balloon model; Buxton et al., 1998; Friston et al, 2003; Stephan et al., 2007) and basically corresponds to a generalised (nonlinear) convolution. In this paper, we will focus exclusively on the neuronal model, because the hemodynamic part is exactly the same as described previously (Stephan et al., 2007). Although we will focus on neuronal systems, the following arguments apply to any complex distributed system with coupled nonlinear dynamics. This means that the procedures described later could (in principle) be applied in different domains.

This material that follows is a bit abstract and could be skipped by the pragmatic reader. It is presented to make four key points: (i) the dynamics of coupled systems can be summarised with a small number of macroscopic variables that describe their behaviour; (ii) the time constants of these macroscopic dynamics are necessarily greater than those of the underlying macroscopic dynamics; (iii) reducing the dynamics to macroscopic variables necessarily induces fast fluctuations in these variables (cf., system noise) *even if the system is deterministic* and (iv) these fluctuations are analytic (continuously differentiable). The last point is crucial because it renders the model non-Markovian and calls for (inversion) schemes that eschew Markovian assumptions (e.g., Generalised Filtering: Friston et al, 2010; Li et al., in press).

Consider the system generating neurophysiologic time-series. This comprises a set of *n* regions, vertices or nodes, where each node corresponds to a vast number of neurons in a cortical area, source or spatial mode (pattern). We will first assume that the dynamics of neuronal states in one node *ξ* = [*ξ*_1_, *ξ*_2_, …]^*T*^ evolve according to some unknown and immensely complicated equations of motion:

(1) $$\begin{array}{c} \dot{\xi }=f\left(\xi \right)\\ ≜\\ {\dot{\zeta }}_{1}=f_{1}\left(\zeta_{1},\ldots ,\zeta_{N}\right)\\ {\dot{\zeta }}_{2}=f_{2}\left(\zeta_{1},\ldots ,\zeta_{N}\right)\\ {\dot{\zeta }}_{3}=\ldots \end{array}$$

These two equations represent the same dynamics but the first is expressed in terms of the original neuronal states (e.g., transmembrane voltages and conductances) of every neuron in the node, while the second equations are expressed in terms of the amplitude of patterns or modes of the original variables, *U*(*ξ*) = *U*^−^*ξ*. This alternative description can be regarded as a change of variables *ξ* = *Uζ* ⇒ *ζ* = *U*^−^*ξ*. We assume that this mapping *U*^−^ : *ξ* → *ζ* is chosen so that it conforms locally to the generalised eigenvectors *U*(*ξ*) of the Jacobian I=∂f/∂ξ, with eigenvalues $U^{-}IU=\lambda$. The Jacobian describes the stability of flow in state-space; i.e., how quickly flow changes with position. We now appeal (heuristically) to the centre manifold theorem and synergetic treatments of high-dimensional, self-organising systems (Ginzburg and Landau, 1950; Carr, 1981; Haken, 1983); see De Monte et al (2003), Melnik and Roberts, 2004 and Davis, 2006, for interesting examples and applications. Namely, we make the assumption that the eigenvalues $\lambda_{k}=U_{k}^{-}IU_{k}$ associated with each mode *ζ*_*k*_ = *U*_*k*_^−^*ξ* are distributed sparsely; $\lambda_{1}>\lambda_{2}>\lambda_{3}\ldots \in R$. That is, one or a small number of them are near zero, whereas the rest are large and negative. This assumption has additional plausibility for neuronal systems, given their tendency to show self-organised criticality and slowing (Stam and de Bruin, 2004; Shin and Kim, 2006; Suckling et al., 2008; Kitzbichler et al., 2009). Critical slowing means that some modes decay slowly and show protracted correlations over time.

**Fig. 1:**
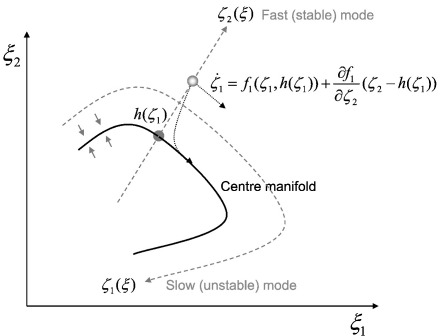
The slaving principle and centre manifolds: This schematic illustrates the basic idea behind the slaving principle. In this example, there are two states, whose flows bring them to an attracting invariant set (the centre manifold); *h*(*ζ*_1_). Once the states have been attracted to this manifold they remain on (or near) it. This means the flow of states can be decomposed into a tangential component (on the manifold) and a transverse component (that draws states to the manifold). This decomposition can be described in terms of a change of coordinates, which implicitly separate fast (stable) transverse dynamics ${\dot{\zeta }}_{2}(\xi )$ from slow (unstable) tangential flow ${\dot{\zeta }}_{1}(\xi )$ on the centre manifold. We exploit this decomposition to motivate the separation of dynamics into a slow, low-dimensional flow on an attracting manifold and a fast (analytic) fluctuating part that describes perturbations away from (and back to) the manifold. Please see the main text for a full description of the equations.

Put simply, all this means is that the dynamics of any system comprising many elements can be decomposed into a mixture of (orthogonal) patterns over variables describing its state. By necessity, some of these patterns dissipate more quickly than others. Generally, some patterns decay so slowly that they predominate over others that disappear as soon as they are created. Mathematically, this means that *P* (principal) eigenvalues *λ*_*p*_ → 0 : *p* ≤ *P* are nearly zero and the associated eigenvectors or modes *U*_*p*_(*ξ*) are slow and unstable. In this case, *ζ*_*p*_ = *U*_*p*_^−^*ξ* : *p* ≤ *P* are known as order parameters. Order parameters are mixtures of states encoding the amplitude of the slow (unstable) modes that determine macroscopic behaviour. Other fast (stable) modes *ζ*_*q*_ = *U*_*q*_^−^*ξ* : *q* > *P* have large negative eigenvalues, which means that they decay or dissipate quickly to an invariant attracting set or manifold, *h*(*ζ*_*p*_), such that ${\dot{\zeta }}_{q}=f_{q}(\zeta_{p},h(\zeta_{p}))=0$. In other words, the invariant (centre) manifold *h*(*ζ*_*p*_) attracts trajectories and contains the solutions to Eq. (1). When there is only one order parameter or principal mode, this manifold is a line or curve in state-space and *ζ*_1_ could represent the distance along that curve (see Fig. 1). The unstable fast modes decay quickly because the eigenvalue is effectively their rate of decay. One can see this easily by taking a first-order Taylor expansion of Eq. (1) about the centre manifold:

(2) $$\begin{array}{c} {\dot{\zeta }}_{q}\approx f_{q}\left(\zeta_{p},h\left(\zeta_{p}\right)\right)+U_{q}^{-}IU_{q}\left(\zeta_{q}-h_{q}\left(\zeta_{p}\right)\right)\\ =\lambda_{q}\left(\zeta_{q}-h_{q}\left(\zeta_{p}\right)\right)\text{.} \end{array}$$

The slow modes (which flow on the centre manifold) are then said to enslave the fast modes (which the centre manifold attracts): This is the “slaving principle” (Ginzburg and Landau, 1950; Carr, 1981; Haken, 1983). The crucial thing here is that the distribution of eigenvalues (rates of dissipation) induces a separation of temporal scales, so that we can approximate Eq. (1) with:

(3) $$\begin{array}{c} {\dot{\zeta }}_{p}=f_{p}\left(\zeta_{p},h\left(\zeta_{p}\right)\right)+\omega_{p}\\ \omega_{p}=\sum_{q}\frac{\partial f_{p}}{\partial \zeta_{q}}\left(\zeta_{q}-h_{q}\left(\zeta_{p}\right)\right)+\ldots \end{array}$$

Here, we have gathered the influences of the fast modes, on the motion of the slow modes, into fast fluctuations $\omega_{p}\in R$ using a Taylor expansion about the centre manifold. We can do this because the states are generally near the centre manifold. Basically, we have thrown away the fast or stable modes and replaced them with fluctuations on the centre manifold. It should be noted that the transverse fluctuations *ζ*_*q*_(*t*) about the centre manifold are not necessarily small. Large fluctuations can occur fairly generically, if periodic orbits embedded in the centre manifold remain transversely unstable. In this case, the transverse dynamics can be of large amplitude, giving rise to what is known as bubbling (Ashwin et al., 1994, 1996).

An important example of the resulting contractions of state-space dynamics are those subtended by symmetries (or near symmetries) among the dynamics, such as the coupling of nearly identical systems (e.g., cortical macrocolumns). In such a setting, the centre manifold approximates a hyper-diagonal subspace or a smoothly mapped (synchronisation) manifold close by (e.g., Hu et al., 2010): The presence of strong transverse flow towards this manifold and a weakly stable or unstable flow on the manifold is exactly the sort of behaviour described by Eq. (3) and has clear relevance for cortical dynamics. Indeed, manifolds that arise from near symmetry in coupled dynamical systems have been studied extensively as models of synchronised neuronal activity (e.g. Breakspear, 2004; Breakspear and Stam, 2005).

Usually, the centre manifold theorem is used to characterise the dynamics on the centre manifold in terms of its bifurcations and structural stability, through normal forms for the associated equations of motion. Here, we are simply using the existence of a centre (or synchronisation) manifold to place formal priors on the form of a generative model. The resulting form (Eq. (3)) comprises slow deterministic dynamics and fast fluctuations that can be treated as analytic (differentiable) random terms. These fluctuations are analytic because they are a mixture of fast deterministic dynamics. Furthermore, in complex self-organising systems, they will exhibit smoothness. This is because some of the fast modes will show critical slowing (i.e., their eigenvalues will move towards zero). This is important because it means Eq. (3) is a random differential equation, not a stochastic differential equation (in which the random terms are Markovian and have no smoothness). Dynamic causal models based on random differential equations call for a slighter more sophisticated treatment than conventional state-space models based on stochastic differential equations (see below).

In summary, we have exploited the separation of temporal scales seen in self-organising dynamics (and the ensuing adiabatic expansion implicit in Eq. 3) to summarise the behaviour of a neuronal ensemble in terms of random differential equations. The key thing about this formulation is that the dynamics of order parameters are much slower than the fluctuations they enslave. We have not considered the exact nature of the order parameters *ζ*_*p*_ but have in mind a single circular (phase) variable (see Fig. 1), such that the rate of change ${\dot{\zeta }}_{1}:=\dot{\zeta }$ reflects the instantaneous frequency of an oscillating mode (cf., Brown et al., 2004; Kopell and Ermentrout, 1986; Penny et al., 2009). If we define $x_{i}:={\dot{\zeta }}^{\left(i\right)}$ as the frequency of the *i*-th node and $\omega_{i}:={\dot{\omega }}^{\left(i\right)}$ as fluctuations in that frequency, Eq. (3) tells us that (dropping the subscript for clarity)

(4) $$\begin{array}{c} {\dot{x}}_{i}={\ddot{\zeta }}^{\left(i\right)}\approx \frac{\partial f^{\left(i\right)}}{\partial \zeta^{\left(i\right)}}{\dot{\zeta }}^{\left(i\right)}+{\dot{\omega }}^{\left(i\right)}\\ =\lambda^{\left(i\right)}x_{i}+\omega_{i}\\ =\omega_{i}\text{.} \end{array}$$

This equation says that, in the absence of exogenous influences, each node will show smooth fluctuations in the frequency at which its principal mode oscillates. Note that the frequency will not decay because the underlying (quasiperiodic) dynamics are on the centre manifold, where *λ*^(*i*)^ = ∂*f*^(*i*)^/∂*ζ*^(*i*)^ = 0 : ∀*i* (there is no imaginary component because there is only one order parameter). One could use either Eq. (3) or Eq. (4) as the formal basis of a generative model for neuronal dynamics. Both share the same key attribute; namely, smooth fluctuations on slow macroscopic dynamics. We will use Eq. (3) as the basis for stochastic DCMs for electromagnetic brain signals in future papers. When modelling fMRI data (in this paper) we will use Eq. (4), because this summary of neuronal activity is the most prescient for fMRI responses. This is because it is generally assumed that fMRI signals scale with the predominant frequency of neuronal activity (Kilner et al., 2005; Laufs and Duncan, 2007; Rosa et al., 2010). We now turn to how different nodes are coupled and see how a separation of fast and slow dynamics in a distributed network of nodes provides a model for network dynamics. We will see that only the slow dynamics are communicated among nodes, which means we can model distributed activity with a small number of macroscopic variables (e.g. one per node) with fast fluctuations that are specific to each node.

### Generative models of network activity

To simplify the model of responses distributed over *n* nodes, we adopt a mean-field assumption (see Deco et al., 2008). This simply means that the dynamics of one node are determined by the mean or average activity in another. Intuitively, this is like assuming that each neuron in one node ‘sees’ a sufficiently large number of neurons in another to render the effective influence the same as the average over all neurons in the source node. The dynamics of these averages are enslaved by the slow modes of other regions so that the motion of the order parameter of each mode is a function of the order parameters from all nodes and local fluctuations:

(5) $$\begin{array}{c} {\dot{\zeta }}^{\left(i\right)}=f^{\left(i\right)}\left(\zeta \right)+\omega^{\left(i\right)}\\ {\ddot{\zeta }}^{\left(i\right)}=\sum_{j=1}^{n}\frac{\partial f^{\left(i\right)}}{\partial \zeta^{\left(j\right)}}{\dot{\zeta }}^{\left(j\right)}+{\dot{\omega }}^{\left(i\right)}\\ \Rightarrow \\ \dot{x}=A\left(\zeta \right)x+\omega \\ \zeta ={\left[\zeta^{\left(1\right)},\cdots ,\zeta^{\left(n\right)}\right]}^{T}\\ x={\left[{\dot{\zeta }}^{\left(1\right)},\cdots ,{\dot{\zeta }}^{\left(n\right)}\right]}^{T}\\ \omega ={\left[{\dot{\omega }}^{\left(1\right)},\cdots ,{\dot{\omega }}^{\left(n\right)}\right]}^{T}\\ A_{\mathrm{ij}}=\frac{\partial f^{\left(i\right)}}{\partial \zeta^{\left(j\right)}}\text{.} \end{array}$$

Here *A* ⊂ *θ* are unknown quantities or parameters encoding the effective connectivity or coupling among nodes. Crucially, this is the random differential equation used in stochastic DCM for fMRI (ignoring bilinear terms and exogenous inputs). In summary, we end up with a very simple model of neuronal dynamics that has been used for many years. In previous work, we motivated the deterministic variant of this model by a Taylor series approximation to unknown non-autonomous dynamics (Friston et al., 2003; Stephan et al., 2008). Here, we have shown how this form emerges naturally from a basic but fundamental principle (the slaving principle), which applies to coupled dynamical systems that self-organise (Ginzburg and Landau, 1950; Haken, 1983). It should be acknowledged that this model is less physiologically grounded than equivalent DCMs for electromagnetic data (where the hidden states are the voltages and currents of neural masses). The hidden neuronal states here are some (unspecified) phase-variable that reports the frequency at which neuronal states orbit an (unspecified) manifold: However, unlike our previous treatments, we have principled reasons to suppose this phase-variable (and its manifold) exist. In previous motivations, we represented the macroscopic behaviour of each node with one (Friston et al, 2003) or two (Marreiros et al, 2008) macroscopic neuronal states; with no motivation for why this was appropriate or sufficient. The current treatment provides that motivation and shows that using a small number of macroscopic states creates fast (analytic) fluctuations, which are ignored in deterministic models. Crucially, these fluctuations are mandated by the slaving principle, even in the absence of stochastic or random effects. This completes our specification of the generative model for distributed neuronal responses under adiabatic and mean field assumptions. We now turn to the inversion of this model, given empirical data.

### Model inversion

In DCM, models are usually inverted by optimising a free-energy bound F(y,q)≤lnp(y|m) on the model log-evidence (or log marginal likelihood of the data *y* conditioned upon a model *m*), assuming the posterior is approximately Gaussian (the Laplace assumption). Optimising this bound, with respect to a proposal density, *q*(*ϑ*), provides two things: First, it provides a free-energy or bound approximation F≈lnp(y|m) to the log-evidence. This will be used in the next section for model comparison or scoring. Second, it makes the proposal density an approximate conditional density q(ϑ)=N(μ,C) on the unknown states and parameters *ϑ* = {*x*, *θ*} of the model, given the data. This conditional density obtains from the construction of the free-energy, which is simply the log-evidence minus the divergence between the proposed and true conditional density. This means that maximising the free-energy minimises the difference between the two, such that the free-energy becomes an approximate log-evidence and the proposal density becomes an approximate conditional density (for technical details see Friston et al., 2003, 2007, 2010).

The key thing that we need to consider here is the nature of the conditional density; in other words, what are the unknown states and parameters, *ϑ* = {*x*, *θ*}? Above, we appealed to the separation of temporal scales (and the slaving principle) to separate the dynamics into a deterministic and a fluctuating part. These fluctuations mean that hidden neuronal states have to be represented probabilistically and call for DCMs that allow for system or state noise (cf. the fluctuations above). Recently, we introduced a Generalised Filtering scheme (Friston et al., 2010) that represents hidden states in generalised coordinates of motion and absorbs unknown (time-invariant) parameters into the filter. This scheme is efficient and allows one to infer on hidden states and parameters using models based on random differential equations like Eq. (5). It accommodates random differential equations by representing the generalised motion of hidden states, which means that their fluctuations are analytic. We will use this scheme in subsequent sections. However, at this point, we note that there is an alternative to Generalised Filtering that uses a deterministic formulation, without random fluctuations. This scheme uses exogenous inputs in deterministic DCMs to model the fluctuations on neuronal states. Essentially, this converts the problem of inferring hidden states into a problem of inferring the parameters (coefficients) of temporal basis functions modelling unknown hidden states (cf. Riera et al., 2004). This rests on reformulating Eq. (4) to give

(6) $$\begin{array}{c} \dot{x}=Ax+\omega \\ =Ax+Cu\\ \omega {\left(t\right)}_{\mathrm{ij}}=\sum_{j}C_{\mathrm{ij}}u{\left(t\right)}_{j}\text{.} \end{array}$$

Here, *u*(*t*)_*j*_ : *j* = 1, …, *J* is the *j*-th temporal basis function. In what follows, we use a discrete cosine basis set, where the number of components is one quarter of the length of the time series. This basis set was chosen because of its well known efficiency in modelling (compressing) typical signals. Note that this deterministic model ensures the fluctuations are smooth and analytic. Under this model, there is no uncertainty about the states, given the parameters *θ* ⊃ {*A*, *C*}, and therefore our conditional density is just over the parameters. In short, the deterministic scheme optimises *q*(*ϑ*) : = *q*(*θ*) by absorbing unknown fluctuations into the parameters, while Generalised Filtering absorbs unknown parameters into the fluctuating states.

Eq. (6) has been introduced to show the formal connection between stochastic and deterministic DCM and promote a particular perspective on exogenous inputs: namely, that they are prior expectations (usually based on known experimental design) about hidden neuronal fluctuations. This perspective is exploited in the last section and provides a graceful link (conceptually and practically) between activation and resting-sate studies; i.e., activation studies can be treated as task-free and *vice-versa*. The only difference is the prior belief we have about the motion of hidden neuronal states. The following comparative evaluations of deterministic and stochastic formulations are not meant to be exhaustive or definitive but are presented to highlight when their formal connection breaks down.

### Stochastic vs. deterministic models

**Fig. 2:**
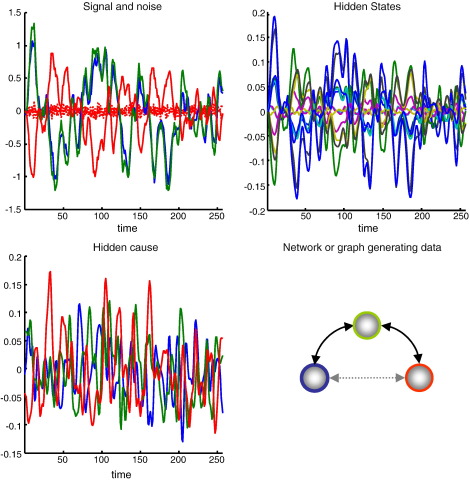
Synthetic data: This figure shows the synthetic data generated by a network or graph with three nodes. The upper left panel shows the simulated activity of each node over 256 (3.22 s) time bins. Signal is shown as solid lines and observation noise as dotted lines. The signal is a nonlinear function of the hidden hemodynamic and neuronal states shown on the upper right. In this model, there are five hidden states per node, which evolve according to the model's equations of motion. The dynamics seen here are caused by random neuronal fluctuations shown in the lower left panel. These were created by convolving a random Gaussian variable with a Gaussian convolution kernel of two bins standard deviation. The ensuing neuronal responses are communicated among nodes by extrinsic connections. In this example, we connected the blue node to the green node and the green node to the red node, as described in the main text. These influences or effective connectivity is denoted by the bidirectional solid black arrows. The light grey arrow denotes a possible but absent edge (anti-edge).

To compare and contrast the stochastic and deterministic schemes, we generated synthetic fMRI data using Eq. (5) and the hemodynamic equations of motion in the forward model of DCM for fMRI (Friston et al., 2003; Stephan et al., 2007). The results of these simulations are shown in Fig. 2 and exhibit the characteristic amplitude and ultra slow fluctuations seen in resting state time-series. This figure shows the response of three nodes, over 256 (3.22 s) time-bins, to smooth neuronal fluctuations that were generated independently for each region. These fluctuations were generated by smoothing a sequence of independent Gaussian variables so that they had a Gaussian autocorrelation function of two time-bins width and a log-precision of four (precision is inverse variance). Small, random fluctuations in the hemodynamic states (like normalised flow, volume and deoxyhemoglobin content) had a Gaussian autocorrelation width of half a time bin and a log-precision of sixteen. These values were chosen to produce a maximum signal change of about 1% in the fMRI signals. The coupling parameters used for this simulation used a small chain of three areas, with reciprocal connections (see black arrows in the insert in Fig. 2):

(7) $$A=\left[\begin{array}{ccc} -.5 & +.4 & 0\\ +.3 & -.5 & -.3\\ 0 & -.2 & -.4 \end{array}\right]$$

**Fig. 3:**
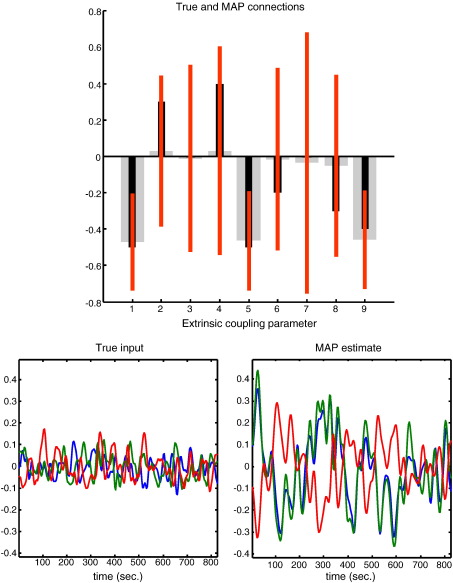
Conditional estimates using a deterministic model: This figure shows the conditional estimates of the coupling parameters among the three nodes of the previous figure. The top panel shows the conditional means (grey bars) and 90% confidence intervals (red bars), superimposed upon the true values (black bars). It can be seen that although the estimates are in the right direction, they are very imprecise (they have a high conditional uncertainty). These estimates were obtained using a deterministic scheme, where unknown (hidden) neuronal causes were modelled as a mixture of temporal basis functions (a discrete cosine set). The true fluctuation or hidden input is shown on the lower left, while the estimated fluctuation is shown on the lower right. This estimate is a reconstitution of the hidden cause, using the conditional estimates of the basis function coefficients. One can see that the amplitude of the input has been overestimated. This reflects the fact that the coupling coefficients were under estimated (upper panel). The colour scheme pertains to the same nodes as in the previous figure.

**Fig. 4:**
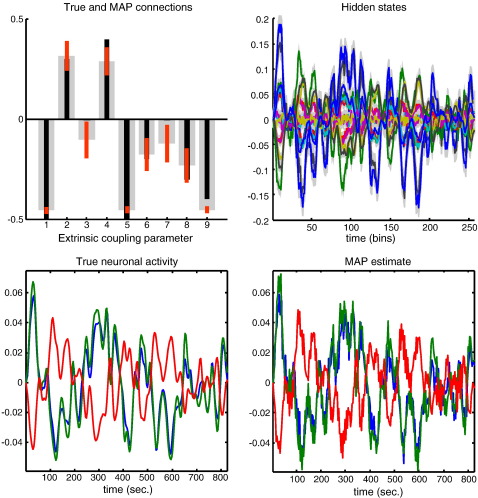
Conditional estimates using a stochastic model: This figure shows similar results to those presented in Fig. 3. However, in this case the conditional estimates were based upon a stochastic model using Generalised Filtering. Here (upper left), we see that the estimates are closer to their true values and are much more precise. Furthermore, the conditional (*maximum a posteriori*; MAP) estimates of the neuronal fluctuations are very close to those elicited by the neuronal input used to simulate the data (compare the left and right lower panels). Because this model includes unknown (hidden) neuronal and physiological states, it also returns a conditional estimate of the hidden states causing responses. These are shown in the upper right panel. The conditional expectations are shown as coloured solid lines and the 90% confidence intervals (tubes) are shown as grey regions. Note that these hidden states are effectively log-states, such that a value of zero corresponds to 100% of the steady-state value. For small deviations from zero, the values of these hidden states correspond roughly to proportional changes. In this example, we see changes of up to about 20% (in blood flow).

The use of positive and negative coupling parameters produces the anti-correlated responses seen between the first two nodes and the third (Fig. 2, upper left panel). The remaining model parameters controlling the hemodynamic convolution (response function) were set to their usual priors (c.f. Friston et al., 2003) and scaled by a small Gaussian deviate with a log-precision of six (i.e., perturbed in a region-specific way by a random scaling with a standard deviation exp(− 6/2) = .0498 of about 5%). These synthetic data were then used for model inversion using (i) a conventional deterministic scheme (Friston et al., 2003) that modelled exogenous input with a discrete cosine set with 64 components and (ii) Generalised Filtering (Friston et al., 2010), respectively. In both schemes, we assumed a prior log-precision of six for observation noise and (for Generalised Filtering) a log-precision of six for hidden neural states and sixteen for the remaining hemodynamic states. This essentially treats neuronal fluctuations as the predominant source of hemodynamics and assumes hemodynamic fluctuations are largely neuronal in origin. The priors on the coupling parameters were mildly informative with a mean of zero and a precision of one half (a variance of two). The ensuing (marginal) conditional densities of the parameters *q*(*A*) for both schemes are shown in Figs. 3 and 4.

Fig. 3 shows the conditional density of the coupling parameters for the deterministic scheme in terms of their 90% conditional confidence intervals (red bars) and true values (black bars). It can been seen that the deterministic scheme, modelling fluctuations with fixed temporal basis functions, underestimates the coupling strengths and consequently overestimates the amplitude of the neuronal fluctuations (represented by exogenous inputs) causing them (i.e., it overestimates the parameters *C* ⊂ *θ*). This is not an invalid result, in that the true values lie within the conditional confidence intervals; however, this model provides inefficient estimates in relation to Generalised Filtering: Fig. 4 shows the equivalent results for Generalised Filtering, which are much more accurate and precise (note the smaller confidence intervals in the upper left panel). In this model, there are no exogenous inputs because dynamics are explained by hidden fluctuations in neuronal and hemodynamic states. Here, the inferred neuronal fluctuations are much closer to the true values used to generate data (lower right panel). Crucially, in contrast to deterministic schemes, stochastic DCM also infers the hidden physiological states that mediate neurovascular coupling (e.g., flow, volume and deoxyhemoglobin content), which are shown in the upper right panel.

Because the deterministic scheme used a discrete cosine set with 64 parameters to model neuronal fluctuations, it implicitly imposes smoothness constraints on the fluctuations. The equivalent smoothness constraints in Generalised Filtering come from priors on the precision of generalised fluctuations (i.e., fluctuations and their high-order temporal derivatives). Interestingly, the computational cost of using the Generalised Filtering scheme was less than the deterministic scheme (about 80 and 100 s per iteration during model inversion, respectively). This was a bit surprising, because Generalised Filtering allows for random fluctuations, not just on the neuronal states, but all (four) hemodynamic states in each node or region (see Fig. 4). In other words, Generalised Filtering was not just estimating the neuronal states but all unknown physiological states. In short, (in this instance) there is little to be gained, either in terms of accuracy, completeness or computational efficiency, from the deterministic formulation. Therefore, we used Generalised Filtering for the rest of this work.

### Summary

In summary, this section has rehearsed the linear approximation to network dynamics used in DCM for fMRI, but from a new perspective. Here, we have taken care to develop this approximation from basic principles (e.g., centre manifold theorem and the slaving principle) and to highlight the role of endogenous fluctuations. These fluctuations model the dynamics attributable to fast (stable) modes that become enslaved by the slow (unstable) modes, which determine macroscopic behaviour. We then used a mean-field assumption to provide a generative model for distributed responses, cast as a random differential equation. One important insight from this motivation is that the time-constants (implicit in the model parameters) of macroscopic network dynamics are much longer than the microscopic time constants (e.g., effective membrane time constants). For example, fluctuations in the characteristic frequency of each mode (Eq. (4)) may be much slower (e.g., 100–10,000 ms) than the oscillatory dynamics (e.g., 10 to 1000 ms) of the (slow) modes themselves, which again are far slower than the dynamics of the fast modes (e.g., .1 to 10 ms). This is important because it suggests that priors on the parameters should allow for slow dynamics. In the next section, we focus on the priors on the effective coupling matrix, *A* ⊂ *θ*, which determines the network dynamics and its architecture.

## Searching model-spaces

Having established the form of the generative model and the optimisation of its parameters, we now turn to optimising the model *per se*. In terms of model optimisation or scoring, we are searching for the model that has the highest evidence or marginal likelihood. Usually, in DCM, one uses the free-energy bound as an approximation to the log-evidence. The problem we now contend with is how to score large numbers of models. For the purposes of network discovery, we can associate each model *m* with a particular sparsity structure on the effective connectivity matrix, *A* ⊂ *θ*. In what follows, we will use several terms from graph theory: A graph comprises a set of nodes and edges (connections), where the edges are deployed according to an *adjacency* matrix A(m). This contains zero or non-zero elements that designate the absence or presence of a connection respectively. In general, graphs can be directed or undirected, cyclic or acyclic. We will deal with directed cyclic graphs. This means that we allow for directed connections and for cycles or loops within the graph; this includes reciprocal connections between two nodes. It is worthwhile noting structural causal modelling based on Bayesian networks (belief networks or directed acyclic graphical models; Spirtes et al, 2000; Pearl, 2009) generally deal with directed acyclic graphs; although there are treatments of linear cyclic graphs as models of feedback (Richardson and Spirtes, 1999). Furthermore, analyses of functional connectivity (and of diffusion tensor imaging data) only consider undirected graphs because the direction of the influence between two nodes is not accessible. This is because functional connectivity is the statistical dependence between two time-series, which has no inherent directionality; although one could argue that directed transfer entropy tests for functional connectivity over time (e.g., Lizier et al., 2010). We can relax these (undirected and acyclic) constraints, because we have an explicit (directed and cyclic) generative model of how data are produced. In what follows, we will consider restrictions on the size of the model-space that is searched, using priors based on each model's adjacency matrix. We then turn to approximate scoring, based upon the conditional densities on the coupling parameters of fully connected graphs from the previous section. Finally, we demonstrate the sensitivity and specificity of the scoring scheme, using simulated data.

### Graphs, priors and dependencies

In this section, we cast network discovery in terms of inference on Bayesian dependency graphs. A Bayesian dependency graph encodes conditional dependencies with edges among variables associated with each node of the graph. The absence of an edge (anti-edge) represents causal independence; i.e., changing the variable in a source node does not change the variable in the target node. This means that discovering the network entails discovering the anti-edges that determine the sparsity structure.

It is important to realise that a dynamic causal model is (formally) a Bayesian dependency graph, in which the form of the dependencies among hidden states is described with deterministic or random differential equations. This means that a DCM can be structurally cyclic (e.g., nodes can be reciprocally connected); however, the underlying Bayesian dependency graph is acyclic. This is because the variables in a parent node change the motion of variables in their children, which can only affect their parent in the future. This precludes instantaneous (cyclic) dependencies. Formally speaking, for every DCM there is an equivalent Dynamic Bayesian Network (DBN), whose nodes represent variables at successive time points. The conditional dependencies among these nodes are specified by the solutions of the differential equations over the discrete time intervals of the DBN. Crucially, the equivalent DBN is acyclic because future variables cannot affect past variables. In short, although a DCM can be structurally cyclic, the implicit dynamic Bayesian network is acyclic.

In the present context, establishing an anti-edge means inferring a DCM (graph) without a particular connection is more likely than the equivalent graph that includes the connection. The implicit difference in log-evidence for these two models is the log-Bayes factor and (by the Neyman–Pearson Lemma) is the most efficient statistic for testing the relative likelihood of both models. This means, in principle, we have a straightforward way to identify conditional dependencies and, by scoring all possible models, discover the underlying dependency graph. Note that this can, in theory, finesse so called missing region problem (c.f., Roebroeck et al., 2009; Daunizeau et al., in press) that can arise when a connection is inferred that is actually mediated by common input. This is because an exhaustive model search will preclude a false inference of conditional dependency between two unconnected nodes, provided the source of common input is part of the full model (and that one can invert it). Furthermore, DCM discovery discloses the underlying network in a way that equivalent analyses of functional connectivity cannot aspire to. This is because functional connectivity is simply the statistical dependence between two nodes that could be conditionally independent when conditioned on a third node. Having said this there are finessed functional connectivity analyses that use partial correlations (e.g., Marrelec et al., 2006, 2009; Smith et al., 2010). Indeed, the principal aim of structural causal modelling (Meek, 1995; Spirtes et al., 2000; Pearl, 2009) is to identify these conditional independencies.

An anti-edge requires that the effective connectivity between two nodes in a DCM is zero. This is enforced by a prior on the unknown coupling parameters, which defines a model. The variances of these priors can be encoded in an adjacency matrix: A prior variance of zero (i.e., no uncertainty) forces the posterior estimate to take its prior mean. Under zero mean priors (as for all coupling parameters in DCM for fMRI), a zero entry in the adjacency matrix thus prohibits an effective connection, establishing an anti-edge between the respective regions. Conversely, a finite value in the adjacency matrix means that the connection has finite variance and that its posterior estimate can take non-zero values. In short, the adjacency matrix from graph theory furnishes formal priors on coupling parameters $A_{\mathrm{ij}}(m)=0\Leftrightarrow p(A_{\mathrm{ij}}|m)=\delta (0)$. This means that there are as many models as there are adjacency matrices. Although, in principle, it should be easy to optimise the model (adjacency matrix) with an exhaustive search, this is seldom possible in practice. This is because the combinatorics of deploying *k* edges among *n* nodes becomes unmanageable when the size of the graph is large.

To finesse this problem we can assume all connections in the brain are directed and reciprocal. This (bidirectional coupling) assumption rests on longstanding anatomical observations (Zeki and Shipp, 1988) that it is rare for two cortical areas to be connected in the absence of a reciprocal connection (there are rare but important exceptions in sub-cortical circuits). More recently, this notion was confirmed in comprehensive analyses of large connectivity databases demonstrating a very strong tendency of cortico-cortical connections to be reciprocal (Kötter and Stephan, 2003). From a functional point of view, modern theories of brain function that appeal to the Bayesian brain, call on reciprocal message passing between units encoding predictions and prediction errors (Mumford, 1992; Friston, 2008). Others theories that rest on reciprocal connections include belief propagation algorithms and Bayesian update schemes that have been proposed as metaphors for neuronal processing (Deneve, 2008). Despite this strong motivation for introducing symmetry constraints on the adjacency matrix, it should be noted that the assumption of reciprocal coupling is not necessary for network discovery; it is used here to demonstrate how prior beliefs can constrain model spaces. Furthermore, this constraint does not mean that the effective connection strengths are identical for both directions of a reciprocal connection: The posterior estimates of coupling can be very different.

**Fig. 5:**
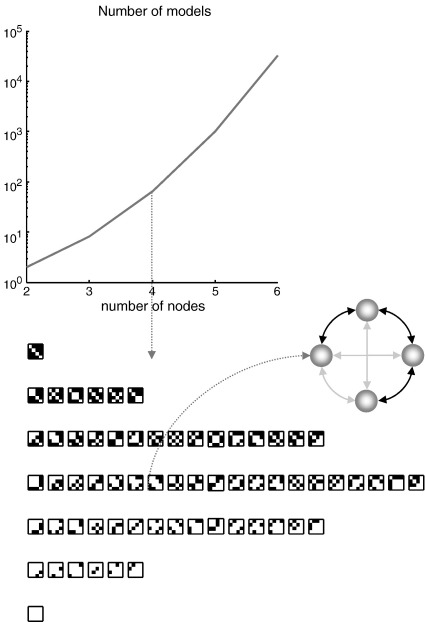
Model spaces and adjacency matrices: This figure illustrates the model spaces induced by considering different adjacency matrices or combinations of edges among the nodes of a graph. The upper panel shows the number of different models that one can entertain as a function of the number of nodes. Here, we placed the additional constraint on the models that each connection has to be bidirectional. The lower panel shows all the alternative models that could be considered, given four nodes. One example is highlighted in the insert, where the solid bidirectional arrows denote edges and the grey arrows denote anti-edges. This particular example was used to generate simulated data for the results described in the next figure.

Even with this constraint, the number of models $|A(m_{i})|=2^{n(n-1)/2}$ can still be too great to explore exhaustively (see Fig. 5). For example, with three regions there are 8 models, for four regions there are 64, for eight regions there are 268,435,456; and so on. This means that there is a combinatoric explosion as one increases the number of nodes in the network. In what follows, we describe a procedure that deals with this problem by scoring models based on the inversion of just one (full) model.

### Approximating the model evidence

We want to find a simple way of scoring large numbers (thousands or millions) of models. We can do this by exploiting the fact that each model can be formed from a fully connected model by switching off various coupling parameters. If we can find a way to approximate the log-evidence of any reduced model (graph), nested within the full model, from the conditional density over the parameters of the full model, then we only need to invert a single model (i.e., the full model) to score an arbitrary number of reduced models extremely efficiently.

More formally, we seek the log-evidence ln *p*(*y*|*m*_*i*_) of model *i*. Here, *m*_*i*_ denotes a reduced model with a subset of (reduced) parameters *θ*_*i*_ ⊂ *θ*_*F*_ that are zero; these define the anti-edges we are trying to discover. By definition, the likelihood of any data under *m*_*i*_ is the same as their likelihood under the full model, given the reduced parameters are zero. This means (via Bayes rule)

(8) $$\begin{array}{c} p\left(y|m_{i}\right)=p\left(y|\theta_{i}=0,m_{F}\right)=\frac{p\left(\theta_{i}=0|y,m_{F}\right)p\left(y|m_{F}\right)}{p\left(\theta_{i}=0|m_{F}\right)}\\ \Rightarrow \\ \ln p\left(y|m_{i}\right)=\ln p\left(\theta_{i}=0|y,m_{f}\right)-\ln p\left(\theta_{i}=0|m_{F}\right)+\ln p\left(y|m_{F}\right)\text{.} \end{array}$$

The last term is just the log-evidence of the full model, which we will treat as zero, because log-evidences are only unique up to an additive constant. Eq. (8) says that the relative log-evidence of a reduced model, given some data, is equal to the log-posterior minus the log-prior that its reduced parameters are zero, under a full model. This is intuitively sensible; in that a conditional density over reduced parameters that is far from a prior of zero suggests the reduced parameters are needed to explain the data. Eq. (8) contains the Savage–Dickey density ratio (Dickey, 1971; see also Friston and Penny, 2011) that is used for nested model comparison, and indeed all classical inference using the extra sum of squares principle (such as F-tests or analysis of variance, ANOVA).

We can approximate the marginal posterior in Eq. (8) using the approximate conditional density q(ϑ|m)=N(μ,C) from the inversion schemes considered in the previous section.

(9) $$\ln p\left(y|m_{i}\right)\approx \ln q\left(\theta_{i}=0|m_{F}\right)-\ln p\left(\theta_{i}=0|m_{F}\right)\text{.}$$

Here *q*(*θ*_*i*_ = 0|*m*_*F*_) is the marginal conditional density over the reduced parameters under the full model. Crucially, after inverting a single (full) model, we can score any new model using Eq. (9). The reason this works is that the new (reduced) model is defined in terms of priors on quantities (parameters) that have been fully characterised during inversion of the full model. Furthermore, Eq. (9) provides an internal test of the quality of the free-energy bound on log-evidence. This is because the relative log-evidences anticipated by Eq. (9) should be the same as those following explicit inversion of each reduced model. In short, we have a way to scan all the models we are interested in and identify the model with the greatest evidence.

To illustrate this *post hoc* model selection we repeated the simulations above using four nodes (with independent neuronal fluctuations in each region) and coupling parameters

(10) $$A=\left[\begin{array}{cccc} -.5 & +.3 & 0 & 0\\ +.3 & -.5 & -.3 & 0\\ 0 & -.3 & -.5 & +.3\\ 0 & 0 & +.3 & -.5 \end{array}\right]\text{.}$$

This corresponds to the coupling architecture (adjacency matrix) illustrated by the insert in Fig. 5 (where the solid black arrows denote edges and the grey arrows denote anti-edges). We then evaluated the log-evidence using Eq. (9), for all (64) models with a prior on the coupling parameters, defined in terms of allowable adjacency matrices

(11) $$\begin{array}{c} p\left(A|m_{i}\right)=N\left(0,\Pi_{i}\right)\\ \Pi_{i}=2\times A\left(m_{i}\right):i=1,\ldots ,64\\ A_{\mathrm{ij}}\in \left\{0,1\right\}\text{.} \end{array}$$

**Fig. 6:**
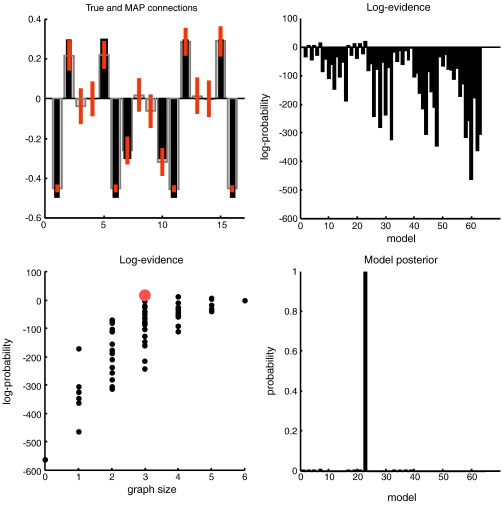
Model inversion and selection: This figure reports the inversion and model selection, following an analysis of simulated data using the graph in the insert of the previous figure. The parameter estimates are shown on the upper left, using the same format as Figs. 3 and 4. It is immediately obvious that the true edges have been detected with reasonably high precision. The conditional density on these coupling parameters was then used to compute the log evidence of (64) reduced models as described in the main text, using the Savage–Dickey ratio. The resulting log-evidence profile over models is shown in the right panels. The upper panel shows the log-evidence as approximated with its free-energy upper bound, while the lower panel shows the corresponding posterior probability over models (assuming flat priors over models). In this example, the correct model has been selected with almost 100% posterior model probability. The log-evidences are also shown as a function of graph size on the lower left. The red dot corresponds to the true model (see previous figure) and has the highest log-evidence. All log-evidences shown in this and subsequent figures are relative to their full model.

The conditional densities of the coupling parameters, following inversion of the full model, are shown in Fig. 6 (upper left panel). As before, these are relatively accurate, with a slightly overconfident underestimate of the (negative) self-connections that had a prior precision of 128. The resulting log-evidences over the 64 models are depicted on the upper right, showing that seven of the reduced models had a greater log-evidence than the full model. Of these, the model with the true architecture had, under flat priors over models, the greatest posterior probability. The lower right panel shows the same results but in terms of model posteriors *p*(*m*_*i*_|*y*) ∝ *p*(*y*|*m*_*i*_) (Eq. (9)). To illustrate the dependency of the log-evidence on the size of each graph (model), we have plotted the log-evidence for each model as a function of its number of (reciprocal) connections (lower left panel). One can see that generally, models with more connections have greater evidence because they provide a more accurate explanation for the data. However, the best model within each graph size shows the opposite behaviour; when the number of connections exceeds the true number, the log-evidence diminishes and always falls below that of the true model (here zero, by definition, and denoted by the red dot). This reflects the fact that model evidence automatically penalises redundant parameters or complexity.

**Fig. 7:**
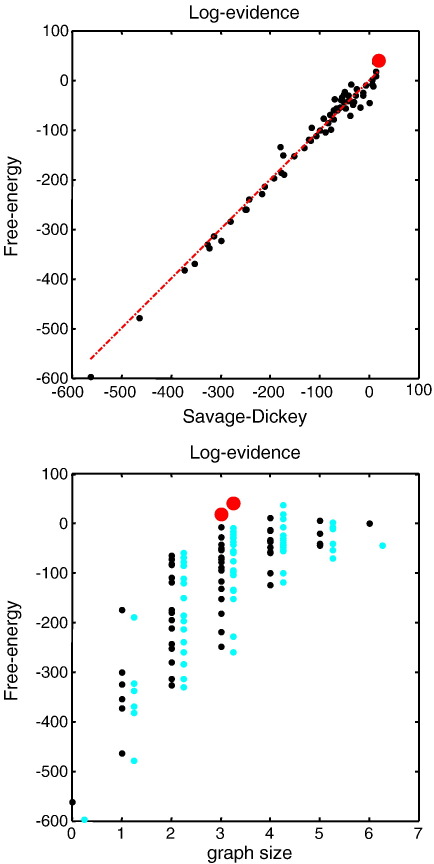
Comparative evaluation of log evidence approximations: This figure presents a comparative evaluation of the *post hoc* log-evidence based upon the conditional density of the full model and the approximation based upon explicit inversions of reduced models. The free-energy of reduced models is plotted against the reduced free-energy in the upper panel and shows a reasonable agreement. The true model is shown as a red dot. The dashed line corresponds to a 100% agreement between the two approximations. The lower panel shows the same data but here as a function of graph size (number of bidirectional edges). The reduced free-energy approximation is shown in black, while the free-energy of reduced models is shown in cyan. Reassuringly, the true model has the highest log-evidence under both proxies, for the correct graph size

To assess the accuracy of the free-energy bound on log-evidence, we explicitly inverted each model and recorded its free-energy. The results in Fig. 7 testify to the quality of the free-energy bound and demonstrate a reasonable correspondence between the proxy in Eq. (9) and the log-evidence as approximated with the free-energy of each reduced model. To achieve this correspondence we had to apply a model prior that penalised each connection by a fixed amount (by subtracting a log-prior cost of 45.8 per connection). Strictly speaking this should not be necessary; however, Generalised Filtering optimises a posterior over parameters that is time-dependent (i.e., optimises the time or path integral of free-energy). This complicates the relationship between posteriors on parameters (which change with time) and priors (which do not). The free-energy used here is therefore based on the Bayesian parameter average over time (see Friston et al., 2010, Appendix 2 for details). Despite this complication, it is reassuring to note that, in models with the correct size, both the *post hoc* and explicit log-evidence proxies identify the same and correct model (see the lower panel of Fig. 7).

### Specificity and sensitivity

**Fig. 8:**
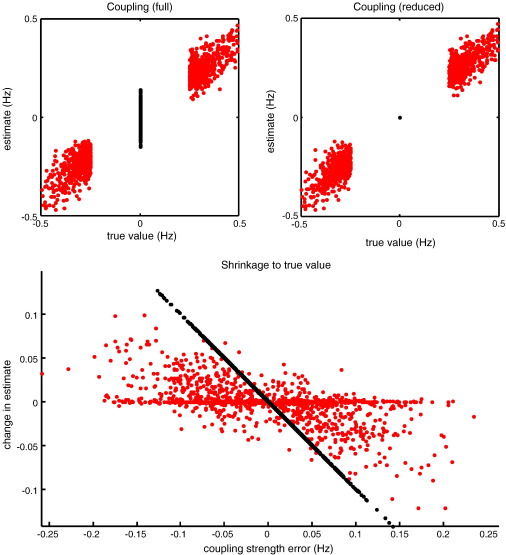
Conditional expectations of the coupling parameters: The upper panels show the conditional expectations of the coupling parameters plotted against their true values for the full model (left) and under the (optimum) reduced model (right). One can see the characteristic shrinkage of a subset of parameters to their prior mean; in this case the parameters associated with anti-edges (black dots). The veracity of this shrinkage depends upon the accuracy of model selection. The lower panel shows the same estimates; however, here, we have tried to highlight that the shrinkage to true values is also evident for the connections that were present (red dots). This panel plots the change in conditional estimate when moving from the full model to the selected (reduced) model against the coupling strength error under the full model. For the connections that were absent, this is simply a straight line, because in all cases, the correct model was chosen. Crucially, we see a similar effect for the connections which were present, with a mild negative correlation between the change in estimate and its mismatch under the full model. Inhibitory self-connections are not included in these results (and did not change much, because they were subject to relatively informative priors).

Finally, we repeated the simulations described above 200 times and recorded the distance between the true model and the model with the largest evidence. Data were generated by sampling coupling parameters from a uniform distribution $A_{\mathrm{ij}}\sim U\left(\frac{1}{4},\frac{1}{2}\right)$ and switching the sign of reciprocal connections randomly. Connections were then eliminated using an adjacency matrix selected at random from the middle row of Fig. 5 (lower panel). Self-connections were sampled from $A_{\mathrm{ii}}\sim N\left(-\frac{1}{2},\frac{1}{4}\right)$. Candidate simulations were discarded if the simulated data exceeded 2% BOLD signal change. The results of these Monte Carlo simulations are shown in Fig. 8: To assess the sensitivity and specificity of the discovery scheme, we used the prior covariance matrix of the model selected to record the number of false positives (when an anti-edge was falsely inferred to be present) and false negatives (when an edge was falsely inferred to be absent). However, this assessment was unnecessary because the model selection procedure attained 100% accuracy. In other words, the correct adjacency structure (model) was selected in all cases and, implicitly, the scheme had a 100% specificity and selectivity for identifying the presence or absence of a connection. This compares favourably with simulations using similar graphs and levels of noise that tested a comprehensive battery of functional connectivity analyses (Smith et al., 2010). Their results show that “in general correlation-based approaches can be quite successful, methods based on higher-order statistics are less sensitive, and lag-based approaches perform very poorly. More specifically: There are several methods that can give high sensitivity to network connection detection on good quality FMRI data, in particular, partial correlation, regularised inverse covariance estimation and several Bayes net methods; however, accurate estimation of connection directionality is more difficult to achieve”. Smith et al. (2010) generated realistic fMRI data using the same type of DCM used here; however, they focussed on the application of functional connectivity methods and (structurally) acyclic graphs. The current results show that it is possible to achieve 100% sensitivity and specificity, with cyclic graphs, provided one uses an appropriate generative model to make inferences about effective connectivity.

The conditional means of individual connections are plotted against their true values (over all simulations) in Fig. 8, for the full model (upper left panel) and selected model (upper right panel). The key thing to note here is the shrinkage of the conditional estimates to the true value of zero, under the optimal model (see the central black dot in the upper right panel). This reflects the fact that this form of model selection implements automatic relevance determination (MacKay, 1995), by virtue of optimising the model evidence with respect to model hyperparameters; in this instance, the shrinkage priors prescribed by an adjacency matrix. Interestingly, there was a mild shrinkage to the true values in the remaining (relevant) connections. This is seen more clearly when plotting the change in conditional estimate against the error (lower panel). One would hope to see that these changes were positive when the error was negative (i.e., the estimate was too high) and *vice versa*. This is exactly what was found (on average).

These results are presented to show that, in principle, it is fairly easy to identify the correct functional architecture of directed cyclic graphs, provided one uses an appropriate generative model and has sufficiently precise data. The data and noise in these simulations had a standard deviation of about .35 and exp(− 4/2) ≈.14, respectively, giving a signal to noise ratio of about 2.6. This is large for a single voxel but not untypical of eigenvariates or averages used to summarise regional activity. For comparison, Smith et al. (2010) used a noise level of .1% to 1% and fMRI signals with maximum amplitudes of about 4%, whereas we used a noise level of .14% and signals with maximum amplitudes of about 2%. For both the simulations and empirical analyses below we used 256 bins of 3.22 s, corresponding to 13.7 min of scanning time.

### Summary

In this section, we have cast network discovery in terms of optimising dynamic Bayesian dependency graphs (represented as DCMs) and considered how this translates into Bayesian model selection. We finessed the problem of searching large model-spaces on two fronts. First, motivated by empirical evidence on anatomical connectivity patterns, we restricted the model-space to bidirectional connections. Although helpful, this constraint is not, strictly speaking, necessary. More importantly, we introduced a proxy scoring scheme based upon the Savage–Dickey density ratio. This works well for the time-series and levels of noise considered. Equipped with this scoring scheme, we can search over enormous model-spaces, while only inverting a single (full) DCM. For a typical fMRI study, model inversion takes about five to ten minutes on a modern computer and *post hoc* model selection takes a few seconds.

It should be remembered that these simulation results show only that it is possible to recover the connectivity structure from realistic responses; however, this clearly rests on having the right generative model. In this section, we used the same model to generate and explain data. In the next section we turn to empirical data, where there is no such guarantee.

## An empirical illustration

In this section, we apply the procedures described in the previous section to an empirical dataset that has been used previously to describe developments in causal modelling and related analyses. We have deliberately chosen an activation study to show that DCM discovery can be applied to conventional studies as well as (design-free) resting-state studies. The interesting distinction between the two applications reduces to prior constraints on the fluctuations. In other words, as discussed in Li et al. (2010), under stochastic DCM, designed or experimental manipulations furnish prior expectations about fluctuations in neuronal states. We can elect to include these priors or ignore them. In the analysis below, we throw these priors away and let the data tell us if our experimental manipulations had any discernable effect upon neuronal activity. We hoped to show that the inferred neuronal states did indeed reflect the experimental manipulations and, at the same time, discover the hierarchical (or non-hierarchical) architecture subtending observed responses. We are not suggesting that this is a good way to analyse activation studies; it just allows us to show the inversion scheme returns valid estimates of hidden states: However, applying stochastic DCM to activation data is potentially interesting, because it allows one to quantify how much neural activity can be attributed to evoked responses (i.e., the experimental design or exogenous inputs) relative to endogenous and recurrent activity. In what follows, we will briefly describe the data used for our analysis and then report the results of network discovery.

### Empirical data

**Fig. 9:**
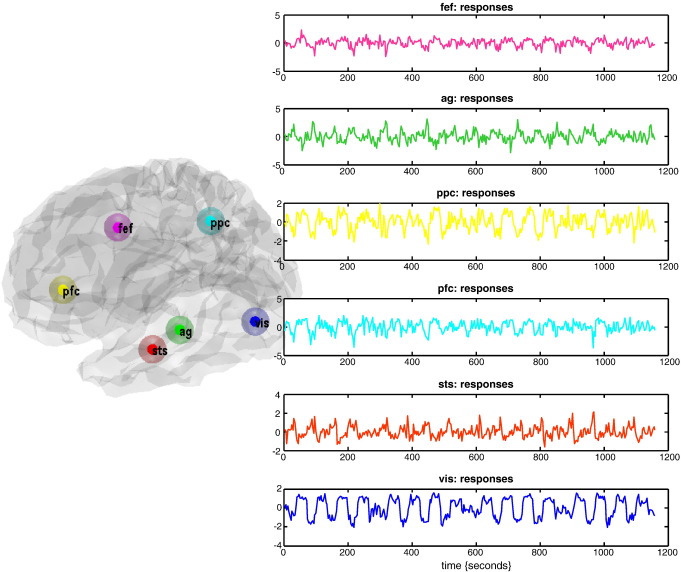
Empirical data: This figure illustrates the data used for the empirical illustration of model selection. Regional summaries were harvested from six regions of interest using the attention to motion paradigm described in the main text. The central location of each region is shown on the left, superimposed on a translucent (canonical) cortical surface in MNI space. The resulting principle eigenvariate (summarising observed responses) are shown in the right panels. In this example, we can see evoked responses in visual areas (every 60 s) with a progressive loss of stimulus-bound activity and a hint of attentional modulation and other fluctuations in higher regions. The predictions of these dynamics, based on inferred hidden states are shown in the next figure.

These data were acquired during an attention to visual motion paradigm and have been used previously to illustrate psychophysiological interactions, structural equation modelling, multivariate autoregressive models, Kalman filtering, variational filtering, DEM and Generalised Filtering (Friston et al., 1997; Büchel and Friston, 1997, 1998; Friston et al., 2003, 2008, 2010; Harrison et al., 2003; Stephan et al., 2008; Li et al., 2010). Data were acquired from a normal subject at two Tesla using a Magnetom VISION (Siemens, Erlangen) whole body MRI system, during a visual attention study. Contiguous multi-slice images were obtained with a gradient echo-planar sequence (TE = 40 ms; TR = 3.22 s; matrix size = 64 × 64 × 32, voxel size 3 × 3 × 3 mm). Four consecutive 100 scan sessions were acquired, comprising a sequence of ten scan blocks of five conditions. The first was a dummy condition to allow for magnetic saturation effects. In the second, *Fixation*, subjects viewed a fixation point at the centre of a screen. In an *Attention* condition, subjects viewed 250 dots moving radially from the centre at 4.7 degrees per second and were asked to detect changes in radial velocity. In *No attention*, the subjects were asked simply to view the moving dots. In a *Static* condition, subjects viewed stationary dots. The order of the conditions alternated between *Fixation* and visual stimulation (*Static*, *No Attention*, or *Attention*). In all conditions subjects fixated the centre of the screen. No overt response was required in any condition and there were no actual changes in the speed of the dots. The data were analysed using a conventional SPM analysis (http://www.fil.ion.ucl.ac.uk/spm). The regions or nodes chosen for network analysis were selected in a rather *ad hoc* fashion and are used here simply to demonstrate procedural details; however, we were careful to avoid the danger highlighted by the analyses of Smith et al. (2010) who note: “…the use of functionally inaccurate ROIs (when defining the network nodes and extracting their associated time series) is extremely damaging to network estimation”. We therefore ensured that the regional summaries were defined functionally by selecting regions showing evoked responses. Six representative regions were defined as clusters of contiguous voxels surviving an (omnibus) *F*-test for all effects of interest at p < .001 (uncorrected) in the conventional SPM analysis. These regions were chosen to cover a distributed network (of largely association cortex) in the right hemisphere, from visual cortex to frontal eye fields (see Table 1 for details). The activity of each region (node) was summarised with its principal eigenvariate to ensure an optimum weighting of contributions for each voxel with the ROI (see Fig. 9). In this example, one can see evoked responses in visual areas (every 60 s) with a progressive loss of stimulus-bound activity and a hint of attentional modulation and other fluctuations in higher regions.

### Model inversion and selection

**Fig. 10:**
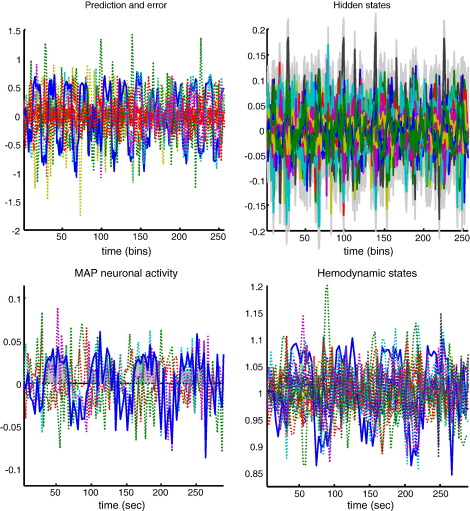
Conditional estimates of hidden states: A summary of the conditional expectations (means) of the hidden states generating observed regional data is shown on the upper right. The solid lines are time-dependent means and the grey regions are 90% confidence intervals (i.e., confidence tubes). These states comprise, for each region, neuronal activity, vasodilatory signal, normalised flow, volume and deoxyhemoglobin content. The last three are log-states. These hidden states provide the predicted responses (conditional expectation) in the upper left for each region and associated prediction errors (red dotted lines), in relation to the observed data. The same data are plotted in the lower panels for about the first four minutes of data acquisition. These results show that the inferred neuronal activity in the visual region (highlighted in blue) follows visual stimulation (grey filled areas — high for attention and low for no attention). The resulting hemodynamic changes are shown as conditional means on the lower right (blue highlights blood flow in the visual region). In this figure log-states have been plotted as states (with a normalised steady-state value of one).

As for the simulated data of the previous section, we inverted a DCM with full connectivity using the first 256 volumes of the time-series. Because we did not know the level of observation noise in these data, we reduced the prior expectation of its log-precision to four; otherwise, the analyses of simulated and empirical data were identical. A summary of the conditional expectations of hidden states generating regional activity are shown in Fig. 10 (upper right). The solid lines are time-dependent means and the grey regions are 90% confidence intervals (i.e., confidence tubes). These states comprise, for each region, neuronal activity, vasodilatory signal, normalised flow, volume and deoxyhemoglobin content, where the last three are log-states. These hidden states provide the predicted responses in the upper left panel for each region and the associated prediction errors (red dotted lines). The same data are plotted in the lower panels for the first four minutes of data acquisition, with hidden neuronal states on the left and hemodynamic states on the right (where log-states are plotted as states). These results are presented to show that inferred neuronal activity in the visual region (highlighted in blue) follows visual stimulation (grey filled areas — high for attention and low for no attention). This confirms that model inversion has effectively deconvolved neuronal activity from hemodynamic signals; and that this deconvolution is veridical, in relation to known experimental manipulations. Recall that the model was not informed of these manipulations but can still recover evoked responses. The associated hemodynamic states of all regions are shown on the lower right (blue highlights blood flow in the visual region). It can be seen that changes in blood flow are in the order of 10%, which is in the physiologically plausible range.

**Fig. 11:**
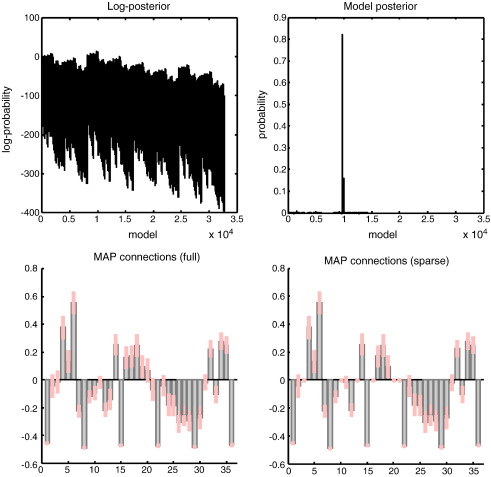
Model selection using empirical data: This figure summarises the results of model selection using the empirical fMRI data. The upper left panel shows the log-evidence profile over the models considered (reflecting different combinations of edges among the six nodes analysed). The implicit model posterior (assuming flat priors over models), is shown on the upper right and suggests that we can be over 80% certain that a particular architecture generated these data. The parameter estimates of the connections under the full (left) and selected model (right) are shown in the lower panels. Again, we see that certain connections have been switched off as the parameter estimates are reduced to their prior value of zero. It is these anti-edges that define the architecture we are seeking. This architecture is shown graphically in the next figure.

Fig. 11 summarises the results of *post hoc* model selection. The inversion of the full model took about 16 min (about 16 iterations of about one minute each), while the *post hoc* search took about 16 s. The upper left panel shows the log-evidence profile over the 2^15^ = 32,768 models considered (reflecting all possible combinations of bidirectional edges among the six nodes analysed). There is a reasonably clear optimum model. This is evident if we plot the implicit log-posterior as a model posterior (assuming flat priors over models), as shown on the upper right. In this case, we can be over 80% certain that a particular network architecture generated the observed fMRI data. The parameter estimates of the connections under the full model (left) and the selected model (right) are shown in the lower panels. One can see that three (bidirectional) connections have been switched off, as their parameter estimates are reduced to their prior value of zero. It is these anti-edges that define the architecture we seek. This is a surprisingly dense network, in which all but three of the fifteen reciprocal connections appear to be necessary to explain observed responses. This dense connectivity may reflect the fact we are using macroscopic regional summaries of activity (that may be engendered by sparse connections on a mesoscopic scale); it may also reflect the fact that we deliberately chose regions that play an integrative (associational) role in cortical processing (c.f., hubs in graph theory; Bullmore and Sporns, 2009). There is an interesting structure to the anti-edges that speaks to the well known segregation of dorsal and ventral pathways in the visual system (Ungerleider and Haxby, 1994): The missing connections are between (i) the superior temporal sulcus and the early visual system, and (ii) the (ventral) superior temporal sulcus/angular gyrus and (dorsal) posterior parietal cortex. On the other hand, there are strong effective connections from the visual system to the prefrontal cortex. This does not mean that there are direct (monosynaptic) connections between these regions; it means they show conditional dependencies that are mediated in a neuronally plausible (polysynaptic) fashion, which cannot be explained by regional activities in the other nodes we considered.

**Fig. 12:**
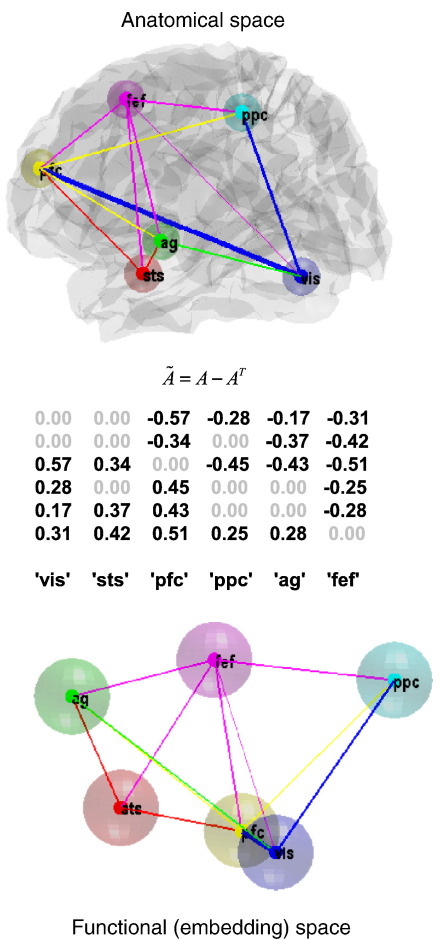
The selected graph in anatomical space and functional space: This figure shows the graph selected (on the basis of the posterior probabilities in the previous figure) in anatomical space and functional (spectral embedding) space. The upper panel shows the same regions depicted in Fig. 9, but now connected using the conditional means of the coupling parameters, under the model selected. The colour of the arrow reports the source of the strongest bidirectional connection, while its width represents its absolute (positive or negative) strength. This provides a description of the architecture or graph in anatomical space. A more functionally intuitive depiction of this graph is provided in the lower panel. Here, we have used spectral embedding to place the nodes in a functional space, where the distance between them reflects the strength of bidirectional coupling. Spectral embedding uses the eigenvectors vectors (principle components) of the weighted graph Laplacian to define a small number of dimensions that best capture the proximity or conditional dependence between nodes. Here, we have used the first three eigenvectors to define this functional space. The weighted adjacency matrix was, in this case, simply the maximum (absolute) conditional estimate of the coupling parameters described in the previous figure. The middle panel shows the asymmetry strengths based on the conditional estimates of the selected model. This provides a further way of characterising the functional architecture in hierarchical terms, based on (bidirectional) coupling.

Fig. 12 shows the underlying graph in anatomical and functional (spectral embedding) space. Note that these plots refer to undirected graphs, although our scheme provides separate estimates for both directions of reciprocal connections (we will look at directed connections strengths below). The upper panel shows the same regions depicted in Fig. 9, but now connected using the conditional means of the coupling parameters, under the reduced (optimal) model. The colour of the arrows reports the source of the strongest bidirectional connection, while its width represents its absolute (positive or negative) strength. This provides a description of the architecture in anatomical space. A more functionally intuitive depiction of this graph is provided in the lower panel. Here, we have used spectral embedding to place the nodes in a functional space, where the distance between them reflects the strength of bidirectional coupling. Spectral embedding uses the eigenvectors *V* = *eig*(*L*) (principal components), of the weighted graph Laplacian, to define locations that best capture the proximity or conditional dependence between nodes. The Laplacian is

(12) $$L_{\mathrm{ij}}=\{\begin{array}{cc} W_{\mathrm{ij}}-\sum_{k}W_{\mathrm{kj}} & :i=j\\ W_{\mathrm{ij}} & :i\neq j \end{array}$$

where *W* is a weighted adjacency matrix based on the conditional expectations of *A* ⊂ *θ*. Fig. 12 uses the first three eigenvectors to define this functional space. This is similar to multi-dimensional scaling but uses the graph Laplacian based upon a weighted adjacency matrix to define similarities. The weighted adjacency matrix was, in this case, simply the maximum (absolute) conditional estimate of bidirectional coupling parameters; *W*_*ij*_ = max(|*A*_*ij*_|, |*A*_*ji*_|).

Spectral embedding suggests that the frontal eye fields (*fef*) play a central and supraordinate role in this network, in the sense that they are remote from the visual region but predominate in terms of the strength of their efferent connections. Interestingly, the prefrontal cortex (*pfc*) and visual region (*vis*) are the furthest apart in anatomical space but the closest pair of nodes in functional space. This reflects the strength of the coupling between these nodes and more generally the tight functional integration between visual and prefrontal areas during visual attention tasks (e.g., Desimone and Duncan, 1995; Gazzaley et al., 2007). Note that this characterisation of the network is insensitive to the sign of connections. Before concluding, we now provide an exemplar analysis that can only be pursued using cyclic directed graphs with asymmetric reciprocal connections; namely an analysis of hierarchical structure.

### Asymmetric connections and hierarchies

Network analyses using functional connectivity or diffusion weighted MRI data cannot ask whether a connection is larger in one direction relative to another, because they are restricted to the analysis of undirected (simple) graphs. However, here we have the unique opportunity to exploit asymmetries in reciprocal connections and revisit questions about hierarchical organisation (e.g., Capalbo et al., 2008; Hilgetag et al., 2000; Lee and Mumford, 2003; Reid et al., 2009). There are many interesting analyses that one could consider, given a weighted (and signed) adjacency matrix. Here, we will illustrate a simple analysis of functional asymmetries: Hierarchies are defined by the distinction between forward (bottom-up) and backward (top-down) connections. There are several strands of empirical and theoretical evidence to suggest that, in comparison to bottom-up influences, the net effects of top-down connections on their targets are inhibitory (e.g., by recruitment of local lateral connections; cf, Angelucci and Bullier, 2003; Crick and Koch, 1998). Theoretically, this is consistent with predictive coding, where top-down predictions suppress prediction errors in lower levels of a hierarchy (e.g., Summerfield et al., 2006; Friston, 2008; Chen et al., 2009). One might therefore ask which hierarchical ordering of the nodes maximises the average strength of forward connections relative to their backward homologue? This can be addressed by finding the order that maximises an asymmetry index, derived from the estimated effective (directed) connection strengths:

(13) $$\begin{array}{c} \alpha =\sum_{i,j<i}{\tilde{A}}_{\mathrm{ij}}\\ {\tilde{A}}_{\mathrm{ij}}=A_{\mathrm{ij}}-A_{\mathrm{ji}}\text{.} \end{array}$$

The resulting order was *vis*, *sts*, *pfc*, *ppc*, *ag*, and *fef*, which is not dissimilar to the vertical deployment of the nodes in functional embedding space (Fig. 12; lower panel). The middle panel shows the asymmetry indices for each connection, based on the conditional estimates of the selected model. This is a pleasing result because it places the visual cortex at the bottom of the hierarchy and the frontal eye fields at the top, which we would expect from the functional anatomy of these regions. Note that there was nothing in the data selection or modelling that could bias the conditional estimates of directed coupling to produce this result. As such, it can be taken as an incidental face validation of the discovery scheme. Before closing, we now turn to a more explicit validation, using empirical data in which conditional dependencies among nodes are destroyed.

### A null analysis

**Fig. 13:**
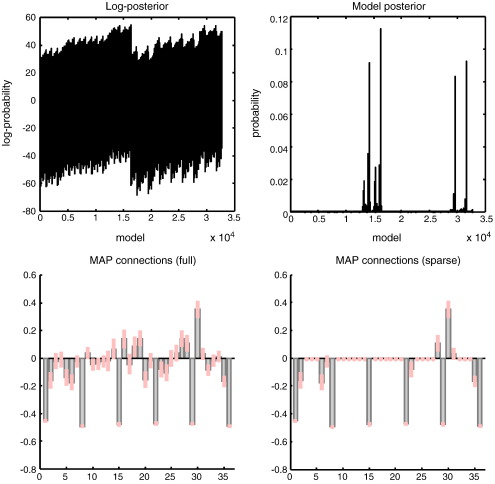
Model selection using null data: This figure has exactly the same format as Fig. 9 but reports *post hoc* model selection results following inversion of phase-shuffled data. Phase shuffling preserves the amplitude and spectral properties of within-node dynamics but destroys any conditional dependencies among nodes. This should result in an edgeless graph, which is nearly the case but not quite.

As a final step towards demonstrating the face validity of the network discovery scheme, we examined whether the discovery scheme detects the absence of conditional dependencies. Conditional dependencies can be destroyed by phase-shuffling the empirical data from the example above to remove any dependencies among nodes, while preserving the within-node dependencies over time (i.e., their spectral properties). Phase-shuffling involves Fourier transforming each regional time-series, randomising the phases (independently in each region) and taking the inverse Fourier transform. Phase-shuffled data only contain evidence for a graph with no edges. Fig. 13 reports the *post hoc* model selection results following inversion of phase-shuffled data using exactly the same format as Fig. 9. This selection should result in an edgeless graph, which is nearly the case but not quite: It can be seen that the log-evidence profile is much shallower in comparison to the analysis of unshuffled data (by an order of magnitude). This results in small model posteriors (upper right) that are distributed over several models. The models with fewer connections are towards the right of these profiles. The model with the greatest evidence retained four out of fifteen connections (see the conditional estimates under the reduced model on the lower right). This is a slightly disappointing result, because we would have hoped to have seen no edges survive model selection. However, there was little evidence for the graph with four connections relative to graphs with fewer connections (with log-Bayes factors of less than three; Kass and Raftery, 1995). In short, even with real data, the *post hoc* model selection proposed for network discovery appears to identify anti-edges, provided one pays attention to the relative evidence for alternative models. Clearly, to assess sensitivity in a classical (frequentist) sense, one would have to assess the distribution of the log-evidence of the most likely model, under the null hypothesis. However, this begs the question: What is the null model for the absence of a conditional dependence or anti-edge?

### Summary

In summary, we have seen how DCM can be applied in a purely data-led way to fMRI studies. In this instance, we used an activation study where we had some prior expectations about the form of the evoked responses. Despite the fact that these expectations were not part of the model, the inferred neural states conformed to what we hoped to elicit experimentally. Furthermore, without biasing inference on models, we disclosed a hierarchical organisation of visual and prefrontal processing areas that has reasonable construct validity in terms of known functional anatomy. A striking result from this data-led application was that the strength of backward connections can be greater than the strength of forward connections: Note all top-down connection from the frontal eye fields were stronger in absolute terms than the equivalent bottom-up connections (Fig. 12). This is entirely sensible, given the greater abundance of backward connections anatomically, both within the cortical hierarchy and from cortex to subcortical structures (e.g., Sillito and Jones, 2002). Furthermore, the importance of backward connections or top-down influences fits comfortably with predictive coding accounts of brain function, which emphasise the importance of predictions that are generated in a top-down fashion (Rao and Ballard, 1999; Friston, 2005).

## Discussion

The quest for discovering causal network structure has a long history, and automatic procedures for determining optimal model structure, given empirical measurements, have played an increasingly important role. For example, various algorithmic search procedures have been proposed for inferring causal structure from association (or covariance) data, often under the framework of Bayesian networks (e.g., Glymour et al., 1987; Spirtes et al, 2000; Pearl, 2009).

In the domain of neuroimaging, there has been a growing interest in searching model-spaces, both in the context of DCM (and other models of effective connectivity) and analyses of functional connectivity. For example, Bullmore et al. (2000) introduced an automatic search procedure for structural equation models of fMRI data, and Valdés-Sosa et al. (2005) has done important work on optimisation of multivariate autoregressive models, in terms of sparsity. Other important work in this area has looked at the efficiency of various correlation schemes and Granger causality, when identifying the sparsity and connectivity structure of real and simulated data (e.g., Cole et al., 2010; Gates et al., 2010; Smith et al., 2010). Finally, discovery of causal network structure from neuroimaging data has also been pursued in the context of Bayesian networks. Ramsey et al. (2010) introduced an “independent multisample greedy equivalence search” algorithm (IMaGES) for fMRI data. This method uses the Bayesian information criterion (BIC; Schwarz, 1978) for automatic scoring of Markov equivalence classes of directed acyclic graphs (DAGs). The restriction to DAGs means, however, that IMaGES only returns acyclic (feed-forward) graphs of effective connectivity.

It is difficult to comment upon the comparative performance of DCM, which deals with dynamic models, in relation to approaches that do not (see Valdés-Sosa et al., 2010 for a full discussion). Other schemes that use dynamic graphs include Granger causality (Granger, 1969) and Dynamic Bayesian Networks (DBN: e.g., Burge et al., 2009; Rajapakse and Zhou, 2007). However, there is a growing appreciation that Granger causality may not be appropriate for fMRI time-series (e.g., Nalatore et al, 2007) and performs poorly in comparison to structural (non-dynamic) approaches based upon partial correlations (Smith et al., 2010). Granger causality and DBN rest on the theory of Martingales (i.e. Markovian assumptions), which may be inappropriate for real dynamical systems, whose fast fluctuations are analytic and may themselves show critical slowing (i.e., non-Markovian or long-memory behaviour) (see Roebroeck et al. (2009) and Friston (2009) for discussion). In fact, one motivation for inventing DCM was to address the shortcomings of autoregressive and underlying Markovian models. Having said this, the computational expediency of functional connectivity and Granger causal schemes mean that they can handle (in principle) vast numbers of nodes and may therefore play a helpful role in identifying candidate networks for the analyses of (directed) effective connectivity described in this paper.

### Future work

Clearly, much work lies ahead in determining the sorts of networks that can be discovered efficiently with the scheme considered here. There are several obvious issues that need exploring: First, we need to establish the level of observation noise that permits veridical discovery: Increasing levels of noise reduces the posterior confidence in non-zero connections and predisposes them to removal during *post hoc* optimisation. The level of noise used in the simulations is not unrealistic but guaranteed a strong connection could be estimated with a high degree of precision. For example, in results of Fig. 6, the difference in log-evidence between the best model and its nearest competitor was about six. This translates into a log-odds ratio of about exp(6) ≈ 400 : 1 or a Z-score of about 2.8. This reflects the efficiency of the model selection and explains why we were able to identify the correct model in all the simulations. We are currently assessing the sensitivity and specificity of *post hoc* model selection as a function of observation noise: The results in this paper can be regarded as proof of principle that it is possible to recover the true network, provided that one has ideal (but not untypical) data. Another key aspect that may determine the identifiability of certain connections is their relative strength and sign. By construction, all the reciprocal connections in our simulations had the same (positive or negative) sign. This is because we found that strong reciprocal connections with opposite signs were estimated inefficiently, with shrinkage to their prior mean of zero. This means that they are unlikely to survive *post hoc* optimisation. One can see heuristically why this occurs (in terms of conditional dependences); however, this and related issues need to be explored properly. Finally, both inversion of the full model and its *post hoc* optimisation are sensitive to the shrinkage priors over the parameters. We used fairly arbitrary (non informative) priors; however, these priors can themselves be optimised using the same formalism behind *post hoc* model optimisation (see Friston and Penny, 2011).

We have illustrated networks with a relatively small number of nodes (two to six). In principle, the scheme can handle much larger networks; however, the time taken to invert the (full) model may become prohibitively long (because the number of free parameters increases quadratically with the number of nodes). Having said this, DCM is used routinely to invert models with thousands of free parameters (e.g. DCM for induced electromagnetic sources; Chen et al., 2008). One approach to large numbers of nodes (e.g., voxels) is to summarise distributed activity in terms of modes or patterns and then estimate the coupling among those patterns (cf, Chen et al., 2008; Havlicek et al., 2010). In terms of the increase in the size of model space with the number of nodes; as noted by one of our reviewers, one could employ a greedy search using the *post hoc* log-evidence. In our current implementation of automatic *post hoc* searches, we eliminate redundant parameters, starting with the eight parameters that have the smallest effect on log-evidence when removed. This process is repeated until no more parameters are removed or less than eight parameters remain. Of course, one would restrict an exhaustive search of models to preclude those that violate prior beliefs.

In this paper, we have modelled all the nonlinearities that cause (chaotic) itinerancy in real biological time-series as fluctuations in random differential equations. Because our DCM is a linear approximation, these nonlinearities are absorbed into the fluctuating terms that are inferred during model inversion. Fluctuations are important because they can predominate in certain contexts. For example, the patterns of synchronisation and coherent activity observed in resting-state time-series (both empirically and in simulations) can themselves wax and wane at a slower timescale. Indeed, it is commonly thought that the ultra slow fluctuations seen in fMRI may reflect a modulation of fast synchronised activity at the neuronal level that may be a principal determinant of observed BOLD signal (Kilner et al., 2005; Deco et al., 2009; de Pasquale et al., 2010). From the point of view of generative models, this suggests that the coupling parameters are themselves state and implicitly time-dependent. One can model this state-dependency, and ensuing itinerancy, by simply adding nonlinear (quadratic) terms to the coupling matrix as described in Stephan et al. (2008). This provides a DCM based on nonlinear random differential equations that can, in principle, be inverted using Generalised Filtering. One of the reasons that we chose the attentional dataset was that we know that there are strong contextual (experimental) effects on the coupling that are usually ascribed to attentional modulation of intrinsic or extrinsic connections in the visual nodes of the network. This modulation has been variously modelled in terms of exogenous (experimental manipulations of attentional set) or endogenous (state-dependent) terms (Li et al., 2010). In future work, we hope to compare models with and without nonlinear (state-dependent) coupling using the inversion and selection schemes described above. In short, network discovery can also be applied to bilinear and nonlinear DCMs to discover functional architectures with nonlinear (state-dependent) effects.

In a similar vein, model averaging and selection procedures currently applied to the free-energy approximations following inversion of reduced models can be applied to the *post hoc* log-evidence used for model discovery (see Stephan et al., 2010 for an overview of these procedures). For example, in group studies (when treating the model as a fixed effect over subjects) one would simply add *post hoc* log-evidences to discover the best model over subjects and proceed in the usual way. Similarly, *post hoc* log-evidences can be used for random effects model selection for group studies. Again we will illustrate this in future application papers.

### Conclusion

In conclusion, we hope to have introduced a scheme that people may find useful when answering questions in a discovery or data-led fashion, while retaining powerful constraints on the way that those data were generated. We have also described a solution to searches on large model-spaces which finesse problems due to combinatorics on connections and computational overhead. We envisage that this approach could be useful in analysing resting-state studies (Damoiseaux and Greicius, 2009; Biswal et al., 2010; Van Dijk et al., 2010) or indeed any data reporting unknown or endogenous dynamics (e.g. sleep EEG). Although we have illustrated the approach using region specific summaries of fMRI data from an activation study, there is no reason why exactly the same approach could not be applied to the activity of distributed modes, such as those from principal or independent component analysis (cf, Havlicek et al., 2010). Finally, having access to the adjacency matrices summarising functional brain architectures (in terms of effective connectivity) opens the door to graph theoretic analyses that leverage important advances in network theory (e.g., Bullmore and Sporns, 2009).

The schemes described in this paper are implemented in Matlab code and are available freely as part of the open-source software package SPM8 (http://www.fil.ion.ucl.ac.uk/spm). Furthermore, the attentional data set used in this paper can be downloaded from the above website, for people who want to reproduce the analyses described in this paper.

## Figures and Tables

**Table 1:** Regions selected for DCM analysis on the basis of an (Omnibus) SPM of the F-statistic testing for evoked responses. Regions are defined as contiguous voxels in the SPM surviving a threshold of p < .001 (uncorrected).The anatomical designations should not be taken too seriously because the extent of several regions covered more than one cytoarchitectonic area, according to the atlas of Talairach and Tournoux.

Name	Rough designation	Location (mm)	Number of (3 mm^3^) voxels
vis	Striate and extrastriate cortex	− 12 − 81 − 6	300
sts	Superior temporal sulcus	− 54 − 30 − 3	269
pfc	Prefrontal cortex	− 57 21 33	48
ppc	Posterior parietal cortex	− 21 − 57 66	168
ag	Angular gyrus	− 66 − 48 21	51
fef	Frontal eye fields	− 33 − 6 63	81
